# Functional Genomics Highlights Differential Induction of Antiviral Pathways in the Lungs of SARS-CoV–Infected Macaques

**DOI:** 10.1371/journal.ppat.0030112

**Published:** 2007-08-10

**Authors:** Anna de Lang, Tracey Baas, Thomas Teal, Lonneke M Leijten, Brandon Rain, Albert D Osterhaus, Bart L Haagmans, Michael G Katze

**Affiliations:** 1 Department of Virology, Erasmus Medical Center, Rotterdam, The Netherlands; 2 Department of Microbiology, School of Medicine, University of Washington, Seattle, Washington, United States of America; University of North Carolina, United States of America

## Abstract

The pathogenesis of severe acute respiratory syndrome coronavirus (SARS-CoV) is likely mediated by disproportional immune responses and the ability of the virus to circumvent innate immunity. Using functional genomics, we analyzed early host responses to SARS-CoV infection in the lungs of adolescent cynomolgus macaques *(Macaca fascicularis)* that show lung pathology similar to that observed in human adults with SARS. Analysis of gene signatures revealed induction of a strong innate immune response characterized by the stimulation of various cytokine and chemokine genes, including interleukin (IL)-6, IL-8, and IP-10, which corresponds to the host response seen in acute respiratory distress syndrome. As opposed to many in vitro experiments, SARS-CoV induced a wide range of type I interferons (IFNs) and nuclear translocation of phosphorylated signal transducer and activator of transcription 1 in the lungs of macaques. Using immunohistochemistry, we revealed that these antiviral signaling pathways were differentially regulated in distinctive subsets of cells. Our studies emphasize that the induction of early IFN signaling may be critical to confer protection against SARS-CoV infection and highlight the strength of combining functional genomics with immunohistochemistry to further unravel the pathogenesis of SARS.

## Author Summary

Severe acute respiratory syndrome coronavirus (SARS-CoV) infection causes a progressive atypical pneumonia. In typical cases, largely confined to adult and elderly individuals, acute respiratory distress syndrome develops, and admission to an intensive care unit is required. Although these complications can be fatal, most SARS patients recover, suggesting that protective immune responses are operational. In this study, we simultaneously examined virus replication and host–response gene expression profiles in macaque lungs during the acute phase of SARS to gain more insight into the early events that take place after SARS-CoV infection. We show that a strong host response is induced in the lungs of SARS-CoV–infected macaques, illustrated by the induction of several pathogenic cytokines and chemokines. Interestingly, antiviral pathways are activated as well, demonstrated by the presence of phosphorylated signal transducer and activator of transcription 1 (STAT1) transcription factors throughout the lung, but not in SARS-CoV–infected cells. A subset of cells was shown to produce interferon-β, a cytokine involved in the resistance to many viral infections and able to activate STAT1. Activation of this antiviral pathway upon SARS-CoV infection may be an important escape route of the host to withstand the devastating effects of SARS-CoV.

## Introduction

Infection with SARS-CoV causes lower respiratory tract disease with clinical symptoms that include fever, malaise, and lymphopenia [1]. Approximately 20%–30% of SARS patients require management in intensive care units, and the overall fatality rate has approached 10%. Interestingly, children seem to be relatively resistant to SARS, but the reason for this restriction is not known [2–4]. The clinical course of SARS follows three phases [5,6]. In the first phase, there is active viral replication and patients experience systemic symptoms. In the second phase, virus levels start to decrease while antibodies, which are effective in controlling infection, increase. However, pneumonia and immunopathological injury also develop in this phase. Ultimately, in the third phase, fatal cases of SARS progress to severe pneumonia and acute respiratory distress syndrome (ARDS), characterized by the presence of diffuse alveolar damage (DAD) [1,7]. It has been hypothesized that the pathological changes are caused by a disproportional immune response, illustrated by elevated levels of inflammatory cytokines and chemokines, such as CXCL10 (IP-10), CCL2 (MCP-1), interleukin (IL)-6, IL-8, IL-12, IL-1β, and interferon (IFN)-γ [8–13]. These in vivo data have been confirmed with in vitro experiments, demonstrating that SARS-CoV infection induces a range of cytokines and chemokines in diverse cell types [14–19].

In contrast, production of type I IFNs seems to be inhibited or delayed by SARS-CoV in vitro [14–18,20–22]. Moreover, no IFN-α or IFN-β has been detected in the sera of SARS patients or in lungs of SARS-CoV–infected mice [23–25]. Recent in vitro studies demonstrated that type I IFN inhibition or delay may be orchestrated by SARS-CoV proteins ORF 3B, ORF 6, and N [26]. The inhibition of IFN production would benefit SARS-CoV replication, since pretreatment of cells with IFN before SARS-CoV infection efficiently prevents replication in these cells [21,27–30]. Furthermore, prophylactic treatment of macaques with pegylated IFN-α reduces SARS-CoV replication in the lungs [31].

Although IFN production was absent in clinical samples, gene and protein expression profiles in these patients were likely impacted by clinical treatments and concurrent preexisting disease. In addition, most if not all virus–host response information is from clinical blood/sera samples that were taken relatively late during infection—little is known about what happens early during infection. Animal studies are of great value to decipher the host's initial innate immune response, without confounding clinical treatment (steroid and mechanical ventilation) or underlying co-morbidity. In order to elucidate early host responses during the acute phase of SARS-CoV infection, we infected cynomolgus macaques with SARS-CoV and used macaque-specific microarrays and real-time (RT)-PCR techniques to study host gene expression profiles. Adolescent cynomolgus macaques infected with SARS-CoV develop DAD similar to SARS patients, but clear most of the virus in the lungs by day 6 [7]. Because SARS-CoV replicates predominantly in the lower respiratory tract of macaques, the virus infects a range of cells, including type 1 and type 2 pneumocytes, that are different from those analyzed in vitro. The ability to simultaneously examine virus replication and host response gene expression profiles in the lungs of these animals during the acute phase of SARS offers the opportunity to further unravel the pathogenesis of SARS.

## Results

### SARS-CoV Replication and Global Gene Expression in Lungs of SARS-CoV–Infected Macaques

Six cynomolgus macaques were inoculated with SARS-CoV strain HKU-39849 and lung tissues were collected at day 1 (*n* = 2, 1A and 1B) or day 4 (*n* = 4, 4A–4D). No lesions or clinical symptoms were detected on day 1 after SARS-CoV infection, whereas on day 4, three out of four monkeys were lethargic, with one of these animals showing mildly labored breathing. Pathological changes at day 4 post infection included DAD, characterized by flooding of the alveoli with edema fluid, infiltration of neutrophils, damage to the alveolar and bronchial epithelia, and occasional type 2 pneumocyte hyperplasia, as described earlier [31]. Four mock-infected animals were included in the study to serve as a reference for host response without viral challenge and to examine outbred inter-animal variation. Our previous experience with A/Texas/36/91 influenza virus demonstrated that viral mRNA was detected in representative samples of the lung rather than throughout the whole lung [32]. Based on this experience, the level of infection in separate lung samples was evaluated using RT-PCR.

**Figure 1:**
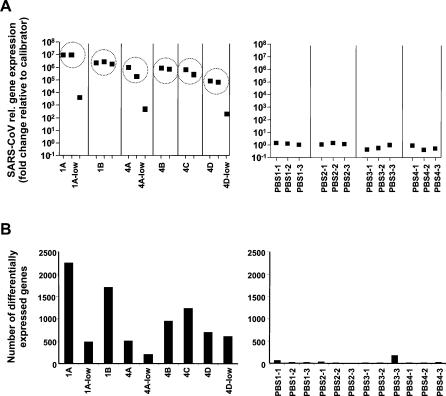
SARS-CoV mRNA Levels and Global Gene Expression in Lungs of SARS-CoV–Infected Macaques (A) RT-PCR was performed on all individual pulmonary samples from SARS-CoV–infected animals and mock-infected animals to determine SARS-CoV levels. Samples from SARS-CoV–infected animals that were pooled for microarray studies are indicated with circles. The three samples with lower levels of virus were hybridized separately. The 12 samples from mock-infected animals (i.e., PBS) were pooled and served as a reference sample. The number after PBS refers to the animal (i.e., PBS 1), while the number after the dash refers to the lung piece (i.e., PBS 1-1). (B) Using microarrays, gene expression in SARS-CoV–infected animals was compared to gene expression in mock-infected animals. The bar graph shows the total number of genes considered to be differentially expressed, defined as an absolute fold change > 2 with *p* < 0.0001. Samples 1A and 1B (day 1) and 4A–4D (day 4) are composed of the pooled individual pulmonary samples shown in (A). Samples 1A low, 4A low, and 4B low are the outliers from (A). Separate mock samples (i.e., PBS) were compared to the total mock pool.

SARS-CoV mRNA was detected in all animals, and 13 pieces out of the total of 16 lung pieces from infected animals contained high levels of virus, while the three remaining pieces of lung contained very low levels of virus (∼3–4 logs lower, Figure 1A). No viral RNA could be detected in the samples from the mock-infected animals. For gene expression experiments, lung samples from SARS-CoV–infected animals were compared to a reference lung sample from mock-infected animals. The three samples with lower virus levels (1A-low, 4A-low, and 4D-low) were analyzed individually so as not to dilute the gene expression of pooled pulmonary samples with higher SARS-CoV levels and also to potentially further define pulmonary infection. Samples from animals with high viral mRNA levels showed greater gene expression changes (∼2,000 genes day 1, ∼800 genes day 4) compared to samples from animals with low levels of viral mRNA (∼400 genes), indicating a response of lung tissue to the virus (Figure 1B). Additionally, the two day 1 animals showed higher numbers of differentially expressed genes than the day 4 animals. In contrast, gene expression analysis of the separate mock samples revealed limited differentially expressed genes.

**Figure 2:**
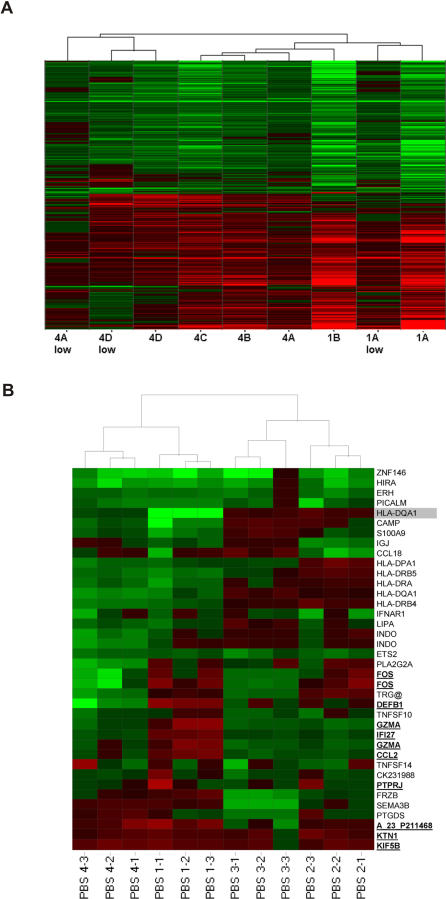
Unsupervised Global Gene Expression Profile of SARS-CoV–Infected Macaques and Individual Variation in Mock-Infected Animals Red corresponds to higher gene expression than that of the controls; green corresponds to lower gene expression. (A) Gene expression profiles result from comparing gene expression in lungs of experimental animals versus gene expression in lungs of mock-infected animals (pooled). Genes were included if they met the criteria of a 2-fold change or more (*p* ≤ 0.0001). A two-of-nine strategy allowed samples to cluster together if profile similarities existed based on timing of inoculation (*n* = 2 samples for day 1). (B) The number after PBS refers to the animal (i.e., PBS 1), while the number after the dash refers to the lung piece (i.e., PBS 1-1). Thirty-eight genes are displayed with an absolute fold change > 2 and *p* <0.0001 in at least two animal samples. Up-regulated genes are indicated in bold underline. Only one gene, *HLA-DQA1*, was down-regulated > 5. No up-regulated genes met these criteria in mock-infected animals. Separate mock samples (i.e., PBS 1-1) were compared to the total mock pool.

In order to examine how gene expression would be influenced by presence of virus, timing after inoculation, and individual animal variation, global expression profiling was performed. Hierarchical clustering methods were used to order rows (genes) and columns (samples) to identify groups of genes or samples with similar expression patterns [33,34]. These data were plotted as a heat map in which each matrix entry represents a gene expression value (Figure 2A). Red corresponds to higher gene expression than that of the controls; green corresponds to lower gene expression. This analysis yielded 2,050 genes with day 1 samples on one side of the heat map and day 4 samples on the other side of the heat map, indicating an influence of timing after inoculation. There are two major roots to the hierarchical dendrogram, with the larger root composed of all the day 1 samples and the three day 4 samples with the highest virus levels. The smaller root is composed of the remaining day 4 samples with the lowest SARS-CoV levels. Although transcriptional profiling shows some variation when comparing samples from the same animal, the underlying gene expression is similar with a reduction in fold change in the “low” samples. These comparisons suggest that both individual animal variation and the “asynchronous” nature of the infection in the animals' lungs are factors involved in determining transcription of cellular genes. To validate that the host response from infected animals comprises a stronger transcriptional profile than individual variation from mock-infected animals, differential gene expression patterns in the separate mock samples were investigated, but only 38 genes were differentially expressed (Figure 2B). These results suggest that underlying basal levels of gene transcription do not confound expression levels after infection. Even in a basal state, some low-level lung-to-lung variations were identified within the same animal but not enough to disrupt segregation of lung pieces based on mock-infected animals.

### Common and Unique Temporal Host Responses to SARS-CoV Infection

**Figure 3:**
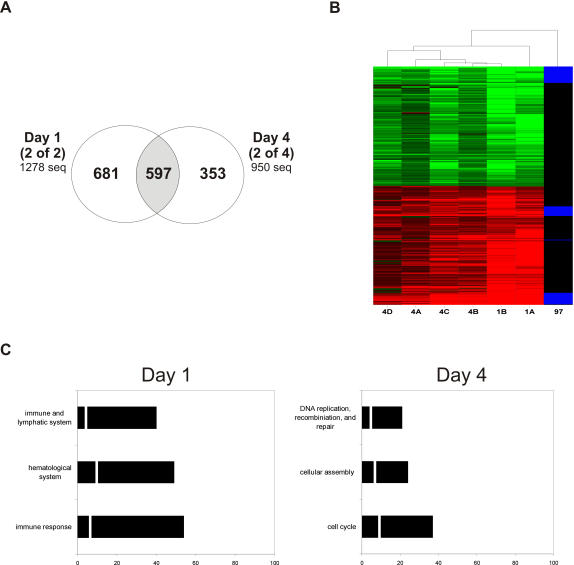
Common and Unique Temporal Gene Responses to SARS-CoV Infection (A) The Venn diagram shows genes with an absolute fold change > 2 and *p* < 0.0001 in two out of the two day 1 samples (left circle) or two out of the four day 4 samples (right circle). (B) The heat map includes the 597 genes from the grey Venn diagram intersections. The right column highlights the 97 genes that are commonly regulated in all six samples with blue. All 97 genes are listed in Figure S1. All gene expression profiles are the results of comparing gene expression in lungs of experimental animals versus gene expression in lungs of mock-infected animals (pooled). (C) Functional annotation was used to categorize the 681 unique genes at day 1, and the 353 unique genes at day 4. The percentage of genes within the top functional categories is indicated in the day 1 and the day 4 bar graph, with a white line indicating the percentage of genes found at the alternative day. Cell-to-cell signaling and cell death, other top categories on day 1, and cell death, cellular growth, and proliferation, additional top categories on day 4, are not included in the bar graph, as these categories were observed to have more genes differentially expressed in the common signature.

In order to elucidate common responses to SARS-CoV throughout the infection as well as unique responses at different time points after inoculation, a Venn diagram was generated with each set (circle) holding to the parameters of an absolute fold change > 2 and *p* < 0.0001 in at least two animals (Figure 3A). The day 1 set contained 1,278 genes and the day 4 set contained 950 genes. When examining host responses that were similar throughout the course of the infection, the intersection of the day 1 and day 4 sets indicates that 597 genes show shared responses. The heat map of these 597 genes is shown in Figure 3B. If more stringent criteria were used to find common responses in all six animals, using the 1,278 genes from the day 1 set and the 129 genes that are differentially expressed in all day 4 animals, a subset of 97 genes was identified. This subset included IFN-stimulated genes (ISGs), like *IFITs*, *MX1*, *GBP1*, and *G1P2*, and also various chemokines and cytokines, such as *CXCL10* (*IP-10*), *CCL2* (*MCP-1*), *IL-6*, and *IL-8* (Figure S1). These same cytokines and chemokines have been reported to be up-regulated in human SARS cases [9–12]. This set also included cathepsin L (*CTSL*), which has been shown to be required for SARS-CoV entry into a cell [35]. Even though only 97 genes were commonly regulated in all animals, indicated with blue bars in Figure 3B, the heat map highlights that the other 500 genes show similar expression trends. Both sets of common-response genes showed similar functionality: cellular growth and proliferation, cell death, cellular movement, immune response, and cell-to-cell signaling.

Next, we analyzed genes that were differentially expressed exclusively on either day 1 or day 4 in order to find signature gene expression patterns for each day. Genes identified as unique responses at day 1 (681 genes) and at day 4 (353 genes) in the Venn diagram showed unique functionality (Figure 3C). The gene expression profile at day 1 shows a prominent innate host response to viral infection; top functional categories on day 1 are the immune response, the hematological system, and the immune and lymphatic system. Genes like *IFN-γ*, *CCL4* (*MIP-1-β*), *CSF3*, *IL1A*, and *TNF* are included in these categories. At day 4, a smaller panel of unique differentially expressed genes that play a role in cell cycle, cellular assembly, and DNA repair were identified like *CCNB2*, *CCNE1*, *CDCA5*, *CENPA*, *CHAF1A*, and *PRC1*.

### Immune Response, Cell Cycle, and Lung Repair Genes with Strongly Induced or Reduced Expression

**Figure 4:**
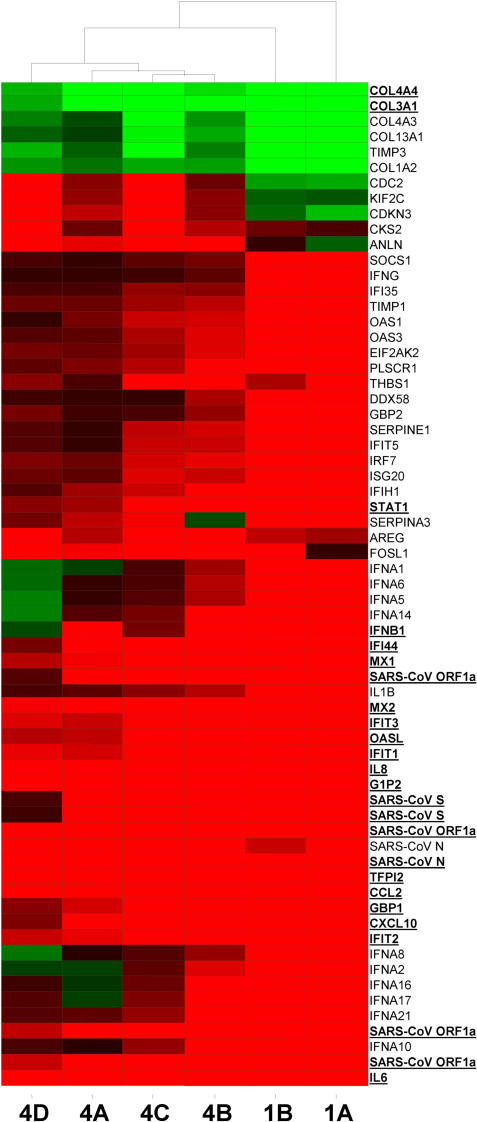
Immune Response, Cell Cycle, and Lung Repair Genes with Strongly Induced or Reduced Expression A selection of genes, involved in the immune response, cell cycle, or lung repair processes, that showed an absolute fold change > 5 and *p* < 0.0001 in both day 1 animals and/or in at least two of the four day 4 animals was made. Genes with an absolute fold change > 5 in both day 1 animals and in at least two of the four day 4 animals are indicated with bold, underlined text. A full summary of genes that show an absolute fold change > 5 and *p* < 0.0001 after SARS-CoV infection is given in Figure S2.

In order to investigate genes that are most strongly regulated after SARS-CoV infection, genes included in the Venn diagram (Figure 3A) that also held to an absolute fold change > 5 were queried (Figure S2). From this set, genes that were involved in the immune response and lung repair processes were used to generate a heat map (Figure 4). A number of genes that have been reported to be up-regulated in SARS patient sera, such as *CCL2* (*MCP-1*), *CXCL10* (*IP-10*), *IL-6*, and *IL-8*, were strongly (∼20-fold) induced in all animals. Many cell cycle and matrix genes indicative of tissue repair processes were also highly differentially expressed at day 4 (e.g., *ANLN*, *AREG*, *CDC2*, *CDKN3*, *CKS2*, *FOSL1*, and *KIF2C*). Likewise, tissue factor pathway inhibitor 2 (*TFPI2*), an anticoagulant, was strongly up-regulated during infection in all animals (averaging ∼20-fold), as well as *PLSCR1*, *SERPINE1* (*PAI1*), and *THBS1*, all genes involved in pro-coagulation and platelet activation, were induced. Concomitant expression of *TFPI2* with these pro-coagulation genes might function as an inhibitory response to restrain the activation of the coagulation pathway during acute inflammation.

Surprisingly, expression of diverse *IFN-α* genes and expression of *IFN-β* was up-regulated ∼10- to 20-fold in the day 1 samples. Furthermore, *IFN-γ*, a type II IFN, was efficiently transcribed on day 1 after SARS-CoV infection (∼5-fold). Other genes associated with the induction of IFNs like *DDX58* (*Rig-I*), *IRF-7*, and signal transducer and activator of transcription 1 (*STAT1*), were also highly induced (∼8-fold). Up-regulation of type I IFNs in these SARS-CoV infected macaques is remarkable, since SARS-CoV inhibits IFN production in many in vitro studies. We did not detect induced *IFN-β* mRNA expression using Ma104 cells or Caco2 cells and the SARS-CoV-HKU virus (unpublished data). Not only IFNs, but also several IFN-responsive genes (e.g., *G1P2*, *GBP1/2*, *IFI/IFITs*, *MX1/2*, *ISG20*, and *OAS1/2/L*) were highly transcribed, showing a persistent activation of the innate immune response. Furthermore, suppressor of cytokine signaling 1(*SOCS1*) is induced at the onset of infection, presumably to establish negative feedback to attenuate cytokine signaling. Of note, *IFIT1* (*ISG56/IFI56*), often used to gauge IFN induction, was up-regulated an average of ∼13-fold.

### Pathogenic and Antiviral Pathways Induced by SARS-CoV

**Figure 5:**
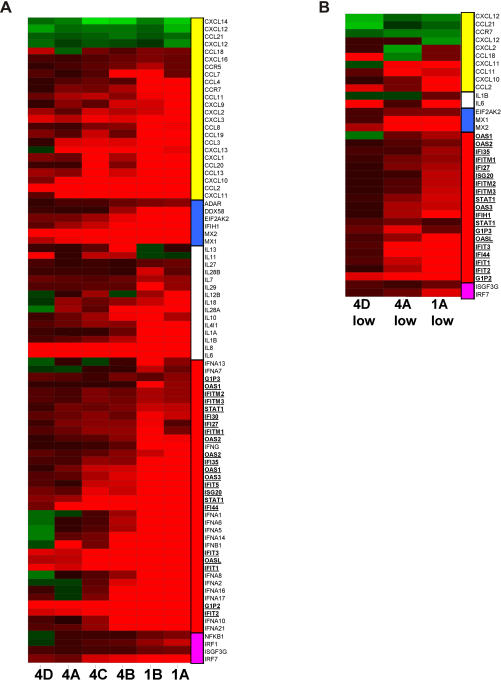
Innate Host Response Profile from Tissues Showing Presence or Absence of Viral mRNA Using a bioset that contained a selection of genes involved in pathological and antiviral pathways, a heat map was created showing all genes with expression changes > 2 and *p* < 0.0001 in at least one of the samples with high SARS-CoV levels (A) or in at least one of the samples with low SARS-CoV levels (B). Although there is some functional overlap with genes, the heat map is segregated by functional annotations. Chemokines (yellow), classical antiviral genes (blue), interleukins (white), JAK/STAT pathway, interferons, or ISGs (red), and transcriptional factors (pink). Genes with an absolute fold change > 5 in two day 1 animals and in at least two of the four day 4 animals are indicated with bold, underlined text.

To further explore some of the pathogenic and antiviral pathways that are induced after SARS-CoV infection, we investigated the transcription of various cytokines, chemokines, IFNs, ISGs, and transcription factors involved in the JAK/STAT pathway. As can be seen in Figure 5A, a wide range of chemokines and cytokines are differentially expressed after SARS-CoV infection in macaque lungs, especially on day 1 after infection. Besides previously mentioned chemokines, we detected monocyte chemotactic protein genes like *CCL8* (*MCP-2*) and *CCL7* (*MCP-3*), but also *CCL11* (eotaxin), a chemotactic protein for eosinophils. In the samples with low SARS-CoV mRNA levels, the induction of chemokines is less evident, suggesting that the presence of these molecules is restricted to areas in the lung where virus is present. Furthermore, SARS-CoV–infected macaques showed a stronger induction of IFNs (14 unique genes) and ISGs (20 unique genes) on day 1 than day 4 and when virus was present at high levels. Note that besides *IFN-α, IFN-β*, and *IFN-γ*, the IFN-λs (*IL-29, IL-28A, IL-28B*), which are type I IFNs, were induced in samples with high SARS-CoV levels. In the absence of viral RNA, no IFNs, but interestingly, a number of ISGs (17 unique genes) were detected, suggesting paracrine stimulation (Figure 5B).

### Confirmation of IL-6, IL-8 IP-10/CXCL10, and IFN-β Expression with RT-PCR and Correlation with SARS-CoV Levels

**Figure 6:**
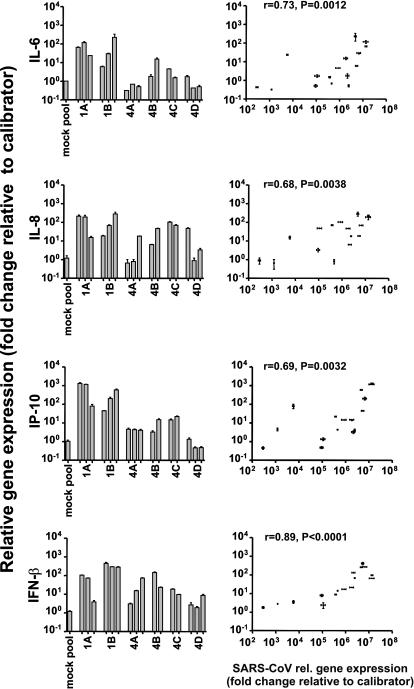
Confirmation of Microarray Results with RT-PCR and Correlation of Induced Genes with Presence of SARS-CoV Quantitative RT-PCR for *IL-6*, *IL-8*, *CXCL10* (*IP-10*), and *IFN-β* was performed on all separate lung samples. Expression levels of these genes were plotted against the presence of SARS-CoV in these samples (as detected by RT-PCR). Correlation coefficients were determined using Spearman's correlation test.

Differential expression of a selection of strongly up-regulated genes, *CXCL10* (*IP-10*), *IL-6*, *IL-8*, and *IFN-β*, was confirmed using RT-PCR (Figure 6). In accordance with the microarray data, the RT-PCR data showed that *CXCL10* (*IP-10*), *IL-6*, *IL-8*, and *IFN-β* were all expressed at levels that were approximately 100 times higher in the SARS-CoV–infected animals at day 1 than in the uninfected control animals and were still elevated on day 4 after infection. As can be seen in Figure 6, the induction of *IFN-β* was strongly correlated to the presence of virus (r_spearman_= 0.88, *p* < 0.0001). For *CXCL10* (*IP-10*), *IL-6*, and *IL-8* the correlation is less evident, which is not surprising since these cytokines can be induced by other factors than the virus itself.

### Detection of IFN-β and Phosphorylated STAT1 in Lung of SARS-CoV–Infected Macaques, Using Immunohistochemistry

**Figure 7:**
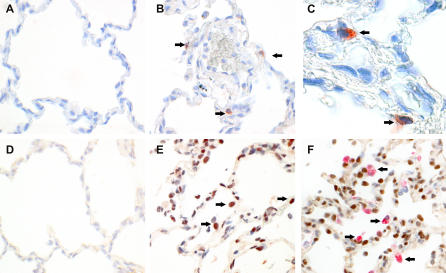
Detection of IFN-β and Phosphorylated STAT1 in Lung of SARS-CoV–Infected Macaques Using Immunohistochemistry (A) Lack of IFN-β expression in lungs of mock-infected macaques (magnification 40×) and (B, C) expression of IFN-β (red) in lungs of SARS-CoV–infected macaques at day 1 post infection (40× [B] and 100× [C], arrowheads). (D) Lack of phosphorylated STAT1 in lungs of mock-infected macaques (40×) and (E) abundant presence of phosphorylated STAT1 (brown) in lungs of SARS-CoV–infected macaques at day 1 post infection (40×, arrowheads). (F) No detection of phosphorylated STAT1 (brown) in SARS-CoV–infected cells (red) (40×, arrowheads).

In order to visualize the host response in the lungs of SARS-CoV–infected macaques, IFN-β production and translocation of phosphorylated STAT1 was studied using immunohistochemistry. In the lungs of the SARS-CoV–infected macaques, a modest number of cells stained positive for IFN-β at day 1 post infection, whereas no IFN-β–positive cells could be detected in mock-infected macaques (Figure 7A–7C). Notably, most of the cells that stained positive for IFN-β were located very close to blood vessels, but not in the alveoli where most SARS-CoV antigen-positive cells (mainly type 2 pneumocytes at 1 day post infection) are located.

To examine whether the IFNs that are produced in the lungs of these SARS-CoV–infected macaques are biologically active and able to induce STAT1 phosphorylation and translocation, lung sections of the infected macaques were stained with antibodies against phosphorylated STAT1. As shown in Figure 7D and 7E, no phosphorylated STAT1 could be detected in the lungs of PBS-infected macaques, while in the lungs of SARS-CoV–infected macaques, cells with phosphorylated STAT1 in their nucleus were abundantly present. Subsequently, the same pieces of lung from SARS-CoV–infected macaques at day 1 were double stained for phosphorlylated STAT1 and SARS-CoV (Figure 7 F). Notably, phosphorylated STAT1 was not detected in the nucleus of SARS-CoV–infected cells (type 2 pneumocytes), while cells directly adjacent to these SARS-CoV–infected cells stained for phosphorylated STAT1 in many, but not all, foci containing SARS-CoV–positive cells. Thus, type I IFNs are produced in the lungs of SARS-CoV–infected macaques, and are able to activate the JAK/STAT pathway. However, translocation of STAT1 does not occur in SARS-CoV–infected pneumocytes.

### Detection of STAT1 Translocation after SARS-CoV Infection In Vitro

**Figure 8:**
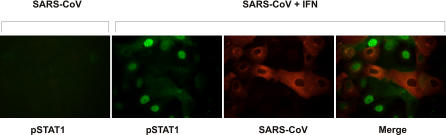
Inhibition of STAT1 Phosphorylation and Nuclear Translocation in SARS-CoV–Infected MA104 Cells MA104 cells were infected with SARS-CoV for 24 h and then either fixed directly (left panel) or treated with type I IFN and then fixed. Subsequently, cells were stained for phosphorylated STAT1 and SARS-CoV. The far right panel shows a merge of the two middle panels, showing an inhibition of STAT1 phosphorylation and translocation in SARS-CoV–infected cells.

Although recent studies indicate that the SARS-CoV ORF6 protein is able to inhibit nuclear translocation of STAT1 in vitro, this was not demonstrated in experiments using infectious SARS-CoV [26]. In order to assess whether SARS-CoV inhibits phosphorylation and translocation of STAT1, MA104 cells were infected with SARS-CoV for 24 h and then either fixed directly or treated with type I IFN. Cells infected with SARS-CoV, but not treated with IFN, stained positive for SARS-CoV (unpublished data), but lacked staining for phosphorylated STAT1, indicating that SARS-CoV or other soluble mediators are not able to induce STAT1 phosphorylation (Figure 8). After treatment of the MA104 cells with IFN, phosphorylated STAT1 could be detected in the nucleus of most cells, but not in the nucleus of SARS-CoV–infected cells (Figure 8). This demonstrates that SARS-CoV inhibits the translocation of phosphorylated STAT1 to the nucleus, confirming our in vivo data. Besides inhibiting translocation of phosphorylated STAT1, SARS-CoV also seems to reduce STAT1 phosphorylation, as the majority of SARS-CoV–infected cells contained low levels of phosphorylated STAT1 in their cytoplasm.

## Discussion

Pathogenic viruses escape the antiviral action of the IFN system by inhibiting both IFN production and signaling pathways. Here, we report that even though production and signaling of type I IFNs is inhibited by SARS-CoV in vitro as well as in SARS-CoV–infected cells in vivo, high levels of type I IFNs are induced in the lungs of SARS-CoV–infected macaques. These IFNs are able to activate STAT1, followed by the transcription of numerous ISGs. Using immunohistochemistry, we revealed that these antiviral signaling pathways were differentially regulated in distinctive subsets of cells. Our results emphasize the strength of combining functional genomics with immunohistochemistry to further unravel the pathogenesis of SARS-CoV infection in cynomolgus macaques.

To our knowledge, this study represents the first functional genomics investigation of SARS-CoV infection in cynomolgus macaques. All experimental animals showed signs of infection because viral mRNA could be detected in random samples from the lung, indicating that the virus had spread throughout the whole lung at the time of necropsy. Furthermore, pathological examination of SARS-CoV–infected macaques at day 4 post infection revealed multifocal DAD [31]. Unlike 10% of humans with SARS, which are mainly restricted to the elderly, adult macaques used in this study do not succumb to SARS-CoV infection. However, the SARS-CoV–induced pathology in these macaques likely resembles the pathological changes seen in the majority of human SARS patients that recover from the disease. Although none of the current animal models has fully reproduced all features of SARS, the most important aspects of this disease are observed in experimentally infected macaques, providing valuable insights into the initial innate immune response after infection without confounding clinical treatment or underlying co-morbidity.

Using macaque-specific microarrays, we were able to observe that with early infection, high levels of viral mRNA corresponded to a strong cellular host response. This strong host response is dominated by genes involved in the immune response and includes a wide range of genes corresponding with what is seen in human ARDS. During the acute phase of human ARDS, activated neutrophils and macrophages enter the alveoli and produce a number of cytokines and chemokines such as IL-6, IL-8, and CXCL10 (IP-10) [36], as were found in the lungs of our SARS-CoV–infected macaques. Researchers have postulated that these genes also predict adverse SARS patient outcome [37]. During the chronic phase of human ARDS, type 2 pneumocytes start to proliferate and differentiate in order to repair the damaged lung. At day 4, the macaque lung shows similar evidence of lung repair, and numerous genes are up-regulated that are involved in cellular growth and proliferation, cell cycle regulation, and DNA replication and repair. Genes involved in cell cycle regulation and proliferation have been previously reported in coronavirus infections other than SARS-CoV and have been characterized by an accumulation of infected cells in the G0/G1 phase [14,38]. We also detected a strong presence of genes involved in the coagulation pathway, including *TFPI2*, *SERPINE1*, and *TIMP1*. The idea of a pro-coagulation profile mimics the clinical-pathological observations of SARS patients that showed unusually disseminated small vessel thromboses in the lungs [5,39]. Additionally, cathepsin L was up-regulated in all SARS-CoV–infected macaques. Induction of this gene after SARS-CoV infection is quite interesting because cathepsin L is an endosomal protease that is necessary for SARS-CoV to infect a cell [35].

Remarkably, SARS-CoV infection in macaques leads to a strong transcription of IFNs. Not only IFN-α, IFN-β, and IFN-λ (all type I IFNs), but also IFN-γ, a type II IFN, were all highly up-regulated, especially on day 1 after infection. The expression of IFN-β, which strongly correlated to the amount of virus present, continued throughout day 4 and was confirmed using immunohistochemistry; IFN-β–positive cells could be detected in the lungs of the SARS-CoV–infected macaques. The induction of IFN-β in these SARS-CoV–infected macaques is surprising, because several reports have shown that SARS-CoV inhibits or delays type I IFN production in a number of cell types [14–18,20,22]. For example, SARS-CoV blocks a step in the activation of IRF-3, a transcription factor that is required for IFN-β induction [21]. In addition, the SARS-CoV proteins ORF3B, ORF6, and nucleocapsid have been shown to function as IFN antagonists, as has the SARS-CoV nsp1 gene that prevents the production of Sendai virus–induced IFN-β in 293 cells [26,40]. Interestingly, it was recently shown that plasmacytoid dendritic cells (pDCs) are able to produce IFN-α and IFN-β after SARS-CoV infection, while conventional DCs did not produce these type I IFNs [41]. pDCs are known for their ability to produce very high amounts of IFN-α and IFN-β and are considered first-line sentinels in immune surveillance in the lung [42–46]. We speculate that the IFN-β–producing cells detected in the lungs of SARS-CoV–infected macaques are pDCs. Future studies may address the nature of these IFN-producing cells once technical difficulties in detecting pDCs in macaque tissues have been tackled. These studies may also shed light on whether decreasing numbers of pDCs observed in clinical blood samples from human SARS patients are caused by sequestering of pDCs by the lungs, destruction of pDCs by SARS-CoV, or destruction or suppression of pDCs by steroid treatment [47].

When IFNs are produced, they bind to their receptors on the cell membrane, after which STAT1, a key member of the JAK/STAT pathway, is phosphorylated and subsequently translocated to the nucleus, followed by the production of a wide range of IFN-stimulated genes. In vitro, SARS-CoV inhibited translocation of STAT1 to the nucleus, and phosphorylation of STAT1 was strongly reduced. However, the inhibition of STAT1 phosphorylation was not absolute because cells with low levels of phosphorylated STAT1 in their cytoplasm were also detected. In accordance with our data, Kopecky-Bromberg et al. recently showed that the SARS-CoV protein ORF6 is able to inhibit STAT1 translocation [26]. This strategy is not unique to SARS. Other viruses have been shown to be able to block signaling of IFNs by affecting phosphorylation and/or translocation of the STAT proteins. For example, measles virus V protein inhibits translocation of STAT1, but does not affect phosphorylation, whereas measles virus P protein blocks both of these processes [48]. Other paramyxoviruses, like Rinderpest virus, Nipah virus, Hendra virus, and mumps virus, as well as flaviviruses like West Nile virus and Japanese encephalitis virus, are able to block activation of STAT1 and STAT2 [49–52]. Inhibition of STAT1 phosphorylation is not always complete. For example, Sendai virus suppresses tyrosine phosphorylation of STAT1 during the early stages of infection, but this block becomes leaky after a couple of hours with phosphorylated STAT1 accumulating in the cytoplasm [53].

In contrast to these in vitro data, we observed phosphorylated STAT1 in the nuclei of numerous cells in the lungs of SARS-CoV–infected macaques, indicating that these cells had been activated by the IFNs produced in the lung. However, phosphorylated STAT1 was not detected in SARS-CoV–infected cells. The observations made in this study indicate that SARS-CoV–infected macaques produce IFNs in response to virus infection and are further capable of activating the STAT1 pathway in cells surrounding the SARS-CoV–infected cells.

The importance of IFNs in controlling SARS-CoV infection has been suggested in several animal studies. Mice clear SARS-CoV in the absence of NK cells, T cells, or B cells, suggesting that innate immune responses are sufficient to limit SARS-CoV infection in these animals [23]. Indeed, STAT1 knock out mice, which are resistant to the effects of IFNs, to some extent show a worsening of pulmonary disease and an increase in viral replication in the lungs compared to normal mice after infection with SARS-CoV [54]. Although IFN treatment was not conducted in SARS-CoV infection mouse studies, prophylactic treatment of macaques with pegylated IFN-α protects type 1 pneumocytes from infection with SARS-CoV [31]. In addition, potent antiviral activity is observed in vitro when cells are treated with IFNs before they are infected with SARS-CoV [27,29,30]. Although we cannot determine the effect of neutralizing IFN-β in SARS-CoV–infected animals, based on the experiments utilizing recombinant IFNs in these animals, we postulate that type I IFNs are partly responsible for the relatively mild clinical symptoms that are seen in SARS-CoV–infected macaques. In addition, a recent study again demonstrated the importance of IFNs in viral infections, as macaques infected with the highly pathogenic and fatal 1918 influenza virus showed limited induction of type I IFNs (only IFNA4 reached fold changes > 5) and delayed induction of ISGs, while macaques infected with the low-pathogenic K173 influenza virus showed a strong induction of these antiviral molecules early during infection [55]. Notably, IFN-β was not up-regulated (absolute fold change < 2) in any of the influenza virus–infected animals, even in those animals that recovered, unlike SARS-CoV–infected macaques that showed a very strong presence of IFN-β.

In conclusion, our study demonstrates that cynomolgus macaques can be infected with SARS-CoV, as indicated by presence of viral mRNA at different locations throughout the lung at day 1 and day 4, with gross pathology becoming noticeable at day 4. Furthermore, we show that infection of cynomolgus macaques with SARS-CoV leads to a strong immune response, including the induction of various cytokines and chemokines, resembling the host response seen in human SARS patients. Strikingly, despite the fact that SARS-CoV infection blocks the production of IFNs in vitro, type I IFNs are strongly induced in the lungs of SARS-CoV–infected macaques. The production of IFN early during infection leads to widespread activation of STAT1 and the production of ISGs. This suggests that, although SARS-CoV blocks IFN signaling in infected cells, locally produced IFNs are capable of activating non-infected cells and possibly can prevent infection of these cells. Thus, SARS-CoV infection in macaques leads to the differential activation of both pathogenic and antiviral signaling pathways in vivo, and the outcome may be determined by the relative contribution of these signaling pathways.

## Materials and Methods

### SARS-CoV infection.

Six cynomolgus macaques *(Macaca fascicularis)* were infected intratracheally with 1 × 10^6^ TCID_50_ SARS-CoV (HKU-39849) as described earlier [31]. Virus stocks were generated in Vero E6 cells that were defective in IFN production. Two animals were euthanized on day 1 after infection and four animals were euthanized on day 4. In addition, four animals were mock (PBS) infected and euthanized on day 4, serving as a negative control group. One lung from each monkey was fixed in 10% formalin for histopathology and immunohistochemistry while the other was used for real-time PCR and microarrays. Lung samples were randomly excised from three different lung areas (cranial, medial, caudal) and stored in RNAlater (Ambion, http://www.ambion.com/). Sixteen pieces of lung were taken from the SARS-CoV–infected animals, two to three pieces of lung per animal. Twelve pieces of lung were taken from the mock-infected animals, three pieces of lung per animal. Individual lung samples in RNAlater were transferred to TRIZOL Reagent (Invitrogen, http://www.invitrogen.com/), homogenized using Polytron PT2100 tissue grinders (Kinematica, http://www.kinematica.ch), and then processed to extract RNA. All experiments were executed under a biosafety level 3, and approval for animal experiments was obtained from the Institutional Animal Welfare Committee.

### Oligonucleotide microarray analysis.

Infected macaque lung samples were co-hybridized with a reference mock-infected macaque lung sample on macaque oligonucleotide arrays containing 131 viral probes, corresponding to 26 viruses, and 22,559 rhesus probes, corresponding to ∼18,000 rhesus genes. The reference mock-infected sample was created by pooling equal mass quantities of total RNA extracted from the 12 individual lung pieces from mock-infected animals. An Agilent 2100 bioanalyzer was used to check the purity of the total RNA prior to cRNA probe production with the Agilent Low RNA Input Fluorescent Linear Amplification kit (Agilent Technologies, http://www.agilent.com/). Arrays were scanned with an Agilent DNA microarray scanner, and image analysis was performed using Agilent Feature Extractor Software (Agilent Technologies). Each microarray experiment was done with two technical replicates using dye reversal [56]. All data were entered into a custom-designed database (Expression Array Manager) and analyzed with Resolver 4.0 (Rosetta Biosoftware, http://www.rosettabio.com/) and Spotfire DecisionSite for Functional Genomics (Spotfire, http://www.spotfire.com/). In our data analysis, genes were selected to be included for transcriptional profile based on two criteria: a greater than 99.99% probability of being differentially expressed (*p* ≤ 0.0001) and an expression level change of 2-fold or greater. Ingenuity Pathway Analysis (Ingenuity Systems, http://www.ingenuity.com/) was used to functionally annotate genes according to biological processes and canonical pathways. In accordance with proposed MIAME standards, primary data are available in the public domain through Expression Array Manager at http://expression.microslu.washington.edu/expression/index.html [57].

### Quantitative real-time RT-PCR.

RT-PCR was performed to detect SARS-CoV mRNA and to validate cellular gene expression changes as detected with microarrays. Each reaction was run in triplicate using Taqman 2x PCR Universal Master Mix (Applied Biosystems, http://www.appliedbiosystems.com/) with primers and probe specific for the SARS-CoV nucleoprotein gene [7], or for macaque cellular genes (sequences shown in Table 1). Differences in gene expression are represented as the fold change in gene expression relative to a calibrator and normalized to a reference, using the 2^−ΔΔCt^ method [58]. GAPDH (glyceraldehydes-3-phosphate dehydrogenase) or 18S rRNA were used as endogenous controls to normalize quantification of the target gene. The samples from the mock-infected macaques were used as a calibrator.

**Table 1:**
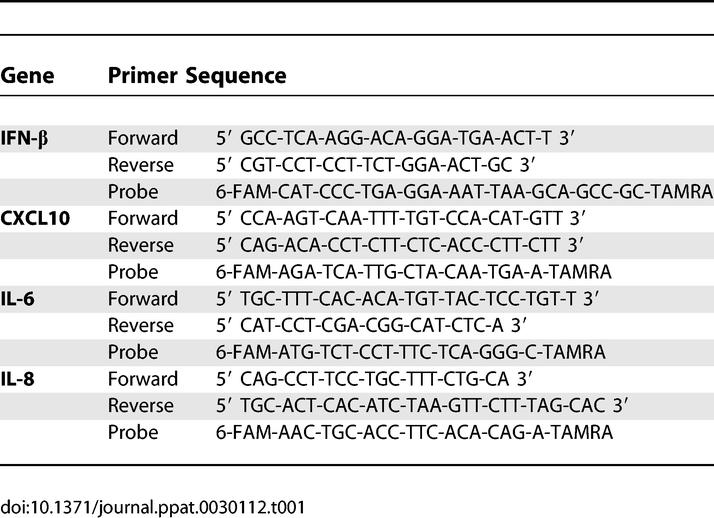
Primers and Probes Used for Quantitative RT-PCR

### Immunohistochemistry.

Formalin-fixed, paraffin-embedded lung samples from SARS-CoV–infected and mock-infected macaques were stained for SARS-CoV, phosphorylated STAT1, and IFN-β using mouse-anti-SARS-nucleocapsid (Clone Ncap4, mouse IgG2b; Imgenex, http://www.imgenex.com/), mouse-anti-phospho-STAT1 (Clone ST1P-11A5, mouse IgG2a-κ; Zymed Laboratories, http://www.invitrogen.com/), and rabbit-anti -IFN-β (Chemicon, http://www.chemicon.com/), respectively. After deparaffinization, antigen retrieval was performed using a citrate buffer for the SARS-CoV and STAT1 staining. No antigen retrieval was performed when staining for IFN-β. Goat-anti-mouse IgG2a HRP, goat-anti-mouse IgG2b AP (Southern Biotech, http://www.southernbiotech.com/), and anti-rabbit IgG-HRP (DAKO, http://www.dako.com/) were used as secondary antibodies. Signals were developed with Fast Red and DAB (Sigma, http://www.sigmaaldrich.com/) and counterstained with Mayer's hematoxylin.

### In vitro SARS-CoV and STAT1 staining.

MA104 cells (African green monkey foetal kidney cells, ECACC) were cultured in Eagle's Minimal Essential Medium (EMEM; Cambrex, http://www.cambrex.com/) supplemented with 2 mM glutamine, 1% non-essential amino acids and 10% foetal bovine serum. Cells were seeded in 96-well plates and infected with SARS-CoV (MOI 0.5), and 24 h after infection, selected wells were treated with universal type I IFN (5,000 U/ml, Sigma) for 30 min at 37 °C. Subsequently, cells were fixed with 10% neutral-buffered formalin and treated with 70% ethanol. SARS-CoV–infected cells were visualized using purified human IgG from a convalescent SARS patient (CSL), followed by staining with an antibody to human IgG, linked to Alexa Fluor 594 (Invitrogen). Phosphorylated STAT1 was visualized using mouse-anti-phospho-STAT1 (Zymed), followed by staining with a FITC-linked antibody to mouse IgG.

## Supporting Information

Figure S1 Heat Map of Common Response Genes That Are Observed in All Animals Depicted are all genes that adhere to an absolute fold change > 2 and *p* < 0.0001 in all animals.
(2.2 MB TIF)

Figure S2 Full Summary of Highly Differentially Expressed Genes (A) Genes with an absolute fold change > 5 and *p* < 0.0001 in both day 1 animals.
(B) Genes with an absolute fold change > 5 and *p* < 0.0001 in two of the four day 4 animals.
(C) Genes with an absolute fold change > 5 and *p* < 0.0001 in both day 1 animals and in at least two of the four day 4 animals. Genes that were used for the heat map in Figure 4 are highlighted in grey.
(5.8 MB TIF)

## Abbreviations

ARDS: acute respiratory distress syndrome
DAD: diffuse alveolar damage
IFN: interferon
IL: interleukin
ISG: interferon-stimulated gene
pDC: plasmacytoid dendritic cell
RT-PCR: real-time PCR
SARS-CoV: severe acute respiratory distress syndrome coronavirus
STAT: signal transducer and activator of transcription
